# Supplementary figures and images for: Defects in the GINS complex increase the instability of repetitive sequences via a recombination-dependent mechanism
Source: PLoS Genet. 2019 Dec 9;15(12):e1008494. doi: 10.1371/journal.pgen.1008494 (PMC6922473; doi:10.1371/journal.pgen.1008494)

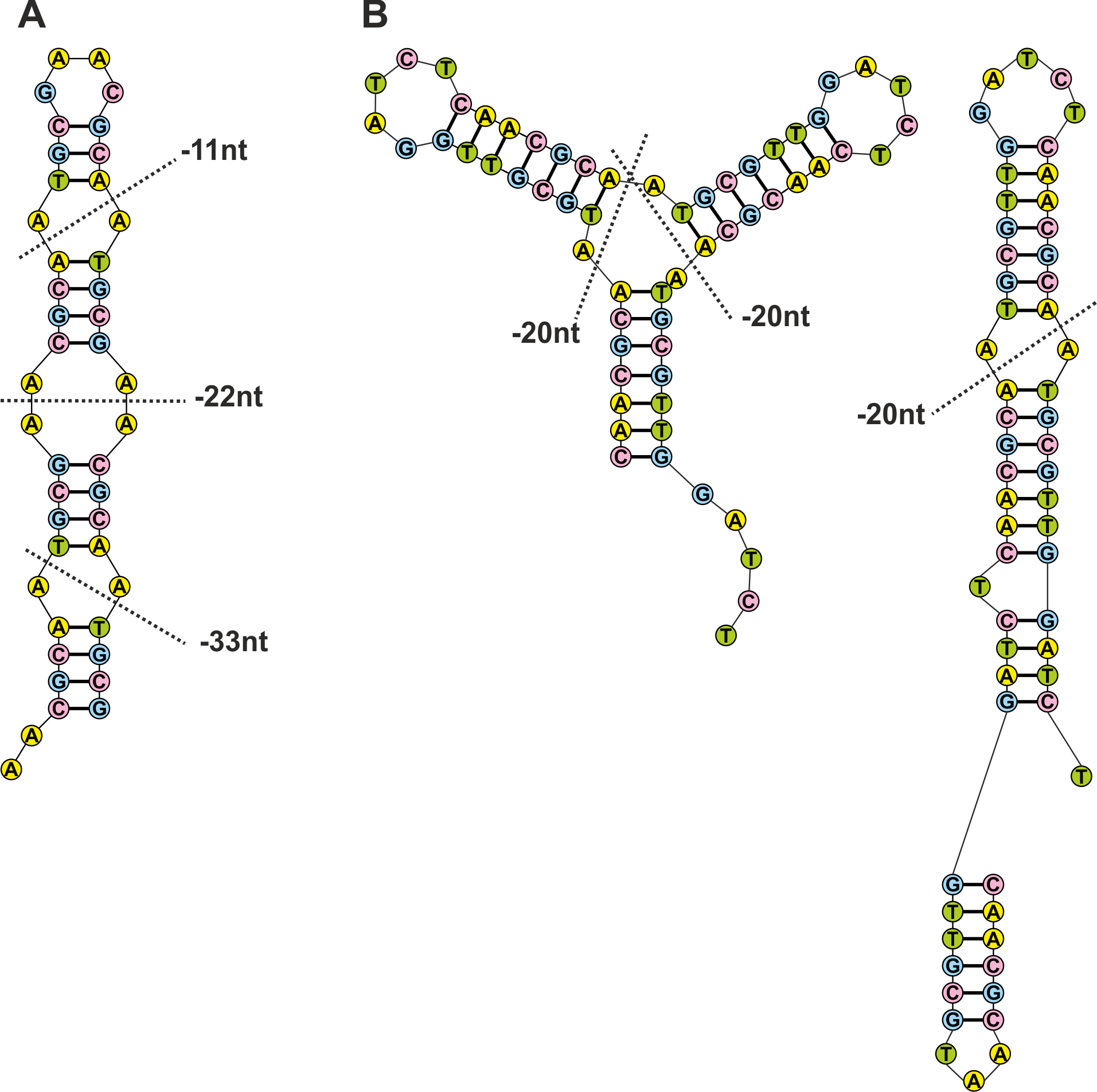

Supplement: S1 Fig — (A) (AACGCAATGCG)4 and (B) (CAACGCAATGCGTTGGATCT)3. Predictions were made using the RNAstructure web server for nucleic acid secondary structure prediction (https://rna.urmc.rochester.edu/RNAstructureWeb/Servers/Predict1/Predict1.html). (TIF) [file pgen.1008494.s001.tif]
