# Supplementary material for: Defects in the GINS complex increase the instability of repetitive sequences via a recombination-dependent mechanism
Source: PLoS Genet. 2019 Dec 9;15(12):e1008494. doi: 10.1371/journal.pgen.1008494 (PMC6922473; doi:10.1371/journal.pgen.1008494)
Supplement: S1 Table — (PDF) [file pgen.1008494.s002.pdf]

S1 Table. *p*-values associated with data presented in Figures 1, 2, 3, 4, 5, 7, 8.

Fig 1

**RS**	**WT**
psf1-1	0,00000003
**GAA**	**WT**
psf1-1	0,00021499
**TTC**	**WT**
**psf1-1**	0,00015708
**CTG**	**WT**
psf1-1	0,00000035

Fig 2

**pKK2**	**WT**
psf1-1	<0,001
**pMD28**	**WT**
psf1-1	<0,001
**p99GT**	**WT**
psf1-1	<0,001
**pMD41**	**WT**
psf1-1	<0,001
**pEAS20**	**WT**
psf1-1	<0,001

Fig 5E-H

**pKK2**	**WT**	***psf1-1***	***rad51Δ***
psf1-1 rad51Δ	0,000108	0,000015	0,005447
rad51Δ	0,000108	0,001049	
psf1-1	0,000108		
**p99GT**	**WT**	***psf1-1***	***rad51Δ***
psf1-1 rad51Δ	0,222248	0,000003	0,000003
rad51Δ	0,000030	0,000001	
psf1-1	0,000103		
**pMD41**	**WT**	***psf1-1***	***rad51Δ***
psf1-1 rad51Δ	0,027487	0,000001	0,113437
rad51Δ	0,002694	0,000002	
psf1-1	0,000089		
**pEAS20**	**WT**	***psf1-1***	***rad51Δ***
psf1-1 rad51Δ	0,109746	0,000148	0,001566
rad51Δ	0,043298	0,000002	
psf1-1	0,000187		

Fig 3A-D

**pKK2**	**WT**	***psf1-1***	***msh2Δ***
psf1-1 msh2Δ	0,000019	0,000005	0,000215
msh2Δ	0,000489	0,000255	
psf1-1	0,000127		
**p99GT**	**WT**	***psf1-1***	***msh2Δ***
psf1-1 msh2Δ	0,000057	0,000022	0,001243
msh2Δ	0,000019	0,000006	
psf1-1	0,000001		
**pMD41**	**WT**	***psf1-1***	***msh2Δ***
psf1-1 msh2Δ	0,000001	0,003708	0,000025
msh2Δ	0,113847	0,000148	
psf1-1	0,000001		
**pEAS20**	**WT**	***psf1-1***	***msh2Δ***
psf1-1 msh2Δ	0,000001	0,583301	0,000075
msh2Δ	0,909850	0,000145	
psf1-1	0,000002		

Fig 7A-D

**pKK2**	**WT**	***psf1-1***	***mms2Δ***
psf1-1 mms2Δ	0,000293	0,022354	0,000907
mms2Δ	0,002622	0,559305	
psf1-1	0,000948		
**p99GT**	**WT**	***psf1-1***	***mms2Δ***
psf1-1 mms2	0,001013	0,000044	0,002551
mms2Δ	0,202023	0,000001	
psf1-1	0,000016		
**pMD41**	**WT**	***psf1-1***	***mms2Δ***
psf1-1 mms2Δ	0,000944	0,000065	0,000065
mms2Δ	0,198450	0,000002	
psf1-1	0,000089		
**pEAS20**	**WT**	***psf1-1***	***mms2Δ***
psf1-1 mms2Δ	0,004039	0,000003	0,000004
mms2Δ	0,039001	0,000000	
psf1-1	0,000261		

Fig 4A-D

**pKK2**	**WT**	***psf1-1***	***rev3Δ***
psf1-1 rev3Δ	0,098578	0,000202	0,006502
rev3Δ	0,017037	0,000032	
psf1-1	0,000001		
**p99GT**	**WT**	***psf1-1***	***rev3Δ***
psf1-1 rev3Δ	0,000049	0,481322	0,000071
rev3Δ	0,758289	0,000108	
psf1-1	0,000076		
**pMD41**	**WT**	***psf1-1***	***rev3Δ***
psf1-1 rev3Δ	0,000237	0,358796	0,000015
rev3Δ	0,803034	0,000042	
psf1-1	0,001874		
**pEAS20**	**WT**	***psf1-1***	***rev3Δ***
psf1-1 rev3Δ	0,000001	0,417430	0,000002
rev3Δ	0,315475	0,000003	
psf1-1	0,000002		

Fig 8A-D

**pKK2**	**WT**	***psf1-1***	***pol32Δ***
psf1-1 pol32Δ	0,000011	0,040005	0,000000
pol32Δ	0,668170	0,000002	
psf1-1	0,000089		
**p99GT**	**WT**	***psf1-1***	***pol32Δ***
psf1-1 pol32Δ	0,000009	0,000000	0,000085
pol32Δ	0,000001	0,000001	
psf1-1	0,000001		
**pMD41**	**WT**	***psf1-1***	***pol32Δ***
psf1-1 pol32Δ	0,000000	0,010812	0,000000
pol32Δ	0,969936	0,000001	
psf1-1	0,000002		
**pEAS20**	**WT**	***psf1-1***	***pol32Δ***
psf1-1 pol32Δ	0,000448	0,000001	0,000051
pol32Δ	0,610385	0,000002	
psf1-1	0,000046		

Fig 5A-D

**pKK2**	**WT**	***psf1-1***	***rad52***
psf1-1 rad52Δ	0,000089	0,000003	0,003704
rad52Δ	0,000089	0,000014	
psf1-1	0,000108		
**p99GT**	**WT**	***psf1-1***	***rad52Δ***
psf1-1 rad52Δ	0,333056	0,000001	0,000055
rad52Δ	0,000129	0,000001	
psf1-1	0,000103		
**pMD41**	**WT**	***psf1-1***	***rad52Δ***
psf1-1 rad52Δ	0,000089	0,000001	0,521711
rad52	0,000239	0,000001	
psf1-1	0,000089		
**pEAS20**	**WT**	***psf1-1***	***rad52Δ***
psf1-1 rad52Δ	0,311603	0,000013	0,003175
rad52Δ	0,118755	0,000012	
psf1-1	0,000129		

Fig 8E-H

**pKK2**	**WT**	***psf1-1***	***pif1Δ***
psf1-1 pif1Δ	0,000300	0,002698	1,000000
pif1Δ	0,000383	0,001455	
psf1-1	0,000948		
**p99GT**	**WT**	***psf1-1***	***pif1Δ***
psf1-1 pif1Δ	0,078984	0,000003	0,490688
pif1Δ	0,053326	0,000002	
psf1-1	0,000016		
**pMD41**	**WT**	***psf1-1***	***pif1Δ***
psf1-1 pif1Δ	0,001435	0,000382	0,000450
pif1Δ	0,027487	0,000002	
psf1-1	0,000089		
**pEAS20**	**WT**	***psf1-1***	***pif1Δ***
psf1-1 pif1Δ	0,000696	0,000002	0,000504
pif1Δ	0,042961	0,000001	
psf1-1	0,000261		
