# Supplementary material for: Defects in the GINS complex increase the instability of repetitive sequences via a recombination-dependent mechanism
Source: PLoS Genet. 2019 Dec 9;15(12):e1008494. doi: 10.1371/journal.pgen.1008494 (PMC6922473; doi:10.1371/journal.pgen.1008494)
Supplement: S2 Table — Contingency table and chi-square test. (PDF) [file pgen.1008494.s003.pdf]

S2 Table. Statistical analysis of Rfa1 foci in Wild-type and *psf1-1* cells presented in Fig 6E. Contingency table and chi-square test.

	Foci number			
	**0**	**1**	**2**	**≥ 3**
**WT**	735	424	115	21
psf1-1	209	301	166	74
**Chi-square statistic**	223,4038			
***p*-value**	3,68891E-48			
