# Supplementary material for: Defects in the GINS complex increase the instability of repetitive sequences via a recombination-dependent mechanism
Source: PLoS Genet. 2019 Dec 9;15(12):e1008494. doi: 10.1371/journal.pgen.1008494 (PMC6922473; doi:10.1371/journal.pgen.1008494)
Supplement: S3 Table — (PDF) [file pgen.1008494.s004.pdf]

S3 Table. Yeast strains used in this work.

Strain	Genotype	Source
SC801	*MAT a CAN1 his7-2 leu2Δ::KanMX4 ura3Δ trp1-289 ade2-1 lys2ΔGG2899-2900* (***PSF1, LEU2***)	[1]
SC802	*MAT a CAN1 his7-2 leu2Δ::KanMX4 ura3Δ trp1-289 ade2-1 lys2ΔGG2899-2900* (***psf1-1, LEU2***)	[1]
SC538	*MAT a CAN1 his7-2 leu2Δ::KanMX4 ura3Δ trp1-289 ade2-1 lys2ΔGG2899-2900* (***PSF1, CaURA3***)	[1]
SC539	*MAT a CAN1 his7-2 leu2Δ::KanMX4 ura3Δ trp1-289 ade2-1 lys2ΔGG2899-2900* (***psf1-1, CaURA3***)	[1]
Y423	SC801 with ***msh2::NAT1***	This work
Y424	SC802 with ***msh2::NAT1***	This work
Y425	SC801 with ***rev3::HPH***	This work
Y426	SC802 with ***rev3::HPH***	This work
Y580	SC801 with ***rad51::HPH***	This work
Y581	SC802 with ***rad51::HPH***	This work
Y582	SC801 with ***mms2::HPH***	This work
Y583	SC802 with ***mms2::HPH***	This work
Y586	SC801 with ***pif1::HPH***	This work
Y587	SC802 with ***pif1::HPH***	This work
Y589	SC801 with ***pol32::HPH***	This work
Y592	SC802 with ***pol32::HPH***	This work
Y595	SC801 with ***rad52::HPH***	This work
Y596	SC802 with ***rad52::HPH***	This work
SC765	MATa CAN1 his7-2 leu2Δ::hisG ura3Δ trp1-289 ade2-1 lys2ΔGG2899-2900	[1]
Y620	MATα CAN1 his7-2 leu2Δ::hisG ura3Δ trp1-289 ade2-1 lys2ΔGG2899-2900	This work
Y621	Y620 with ***msh2::NAT1***	This work
Y622	Y620 with ***rev3::HPH***	This work
Y623	Y620 with ***rad51::HPH***	This work
Y624	Y620 with ***mms2::HPH***	This work
Y625	Y620 with ***pif1::HPH***	This work
Y626	Y620 with ***pol32::HPH***	This work
Y627	Y620 with ***rad52::HPH***	This work
Y631	MATa/α CAN1 his7-2 leu2Δ::hisG/leu2 *Δ::KanMX4* ura3Δ trp1-289 ade2-1 lys2ΔGG2899-2900 ***msh2::NAT1/MSH2*** (***psf1-1, LEU2)/PSF1*** Cross of Y621 and SC802	This work
Y632	MATa/α CAN1 his7-2 leu2Δ::hisG/leu2 *Δ::KanMX4* ura3Δ trp1-289 ade2-1 lys2ΔGG2899-2900 ***rev3::HPH/REV3*** (***psf1-1, LEU2)/PSF1*** Cross of Y622 and SC802	This work
Y633	MATa/α CAN1 his7-2 leu2Δ::hisG/leu2 *Δ::KanMX4* ura3Δ trp1-289 ade2-1 lys2ΔGG2899-2900 ***rad51::HPH/RAD51*** (***psf1-1, LEU2)/PSF1*** Cross of Y623 and SC802	This work
Y634	MATa/α CAN1 his7-2 leu2Δ::hisG/leu2 *Δ::KanMX4* ura3Δ trp1-289 ade2-1 lys2ΔGG2899-2900 ***mms2::HPH/MMS2*** (***psf1-1, LEU2)/PSF1*** Cross of Y624 and SC802	This work
Y635	MATa/α CAN1 his7-2 leu2Δ::hisG/leu2 *Δ::KanMX4* ura3Δ trp1-289 ade2-1 lys2ΔGG2899-2900 ***pif1::HPH/PIF1*** (***psf1-1, LEU2)/PSF1*** Cross of Y625 and SC802	This work
Y636	MATa/α CAN1 his7-2 leu2Δ::hisG/leu2 *Δ::KanMX4* ura3Δ trp1-289 ade2-1 lys2ΔGG2899-2900 ***pol32::HPH/POL32*** (***psf1-1, LEU2)/PSF1*** Cross of Y626 and SC802	This work
Y637	MATa/α CAN1 his7-2 leu2Δ::hisG/leu2 *Δ::KanMX4* ura3Δ trp1-289 ade2-1 lys2ΔGG2899-2900 ***rad52::HPH/RAD52*** (***psf1-1, LEU2)/PSF1*** Cross of Y627 and SC802	This work
Y433	SC801 with **(CTG)x25 from pMA1**	This work
Y434	SC802 with **(CTG)x25 from pMA1**	This work
Y610	SC801 with **(GAA)x25 from pMA5**	This work
Y611	SC802 with **(GAA)x25 from pMA5**	This work
Y612	SC801 with **(TTC)x25 from pMA6**	This work
Y613	SC802 with **(TTC)x25 from pMA6**	This work
Y614	SC801 with **random sequence from pMA7**	This work
Y615	SC802 with **random sequence from pMA7**	This work
BY4741 *rad51Δ*	*MAT a his3Δ1 leu2Δ0 met15Δ0 ura3Δ0 rad51::KanMX4*	Open Biosystems
W3775-12C	*MATa ADE2 bar1::LEU2 trp1-1 LYS2 RFA1-8ala-YFP RAD5*	[2]
Y599	SC538 with ***RFA1-YFP***	This work
Y600	SC539 with ***RFA1-YFP***	This work

1. Grabowska E, Wronska U, Denkiewicz M, Jaszczur M, Respondek A, Alabrudzinska M, et al. Proper functioning of the GINS complex is important for the fidelity of DNA replication in yeast. Mol Microbiol. 2014;92: 659–680. doi:10.1111/mmi.12580
2. Lisby M, Barlow JH, Burgess RC, Rothstein R, Street W, York N. Choreography of the DNA Damage Response : Spatiotemporal Relationships among Checkpoint and Repair Proteins. 2004;118: 699–713. doi:https://doi.org/10.1016/j.cell.2004.08.015
