# Supplementary material for: Defects in the GINS complex increase the instability of repetitive sequences via a recombination-dependent mechanism
Source: PLoS Genet. 2019 Dec 9;15(12):e1008494. doi: 10.1371/journal.pgen.1008494 (PMC6922473; doi:10.1371/journal.pgen.1008494)
Supplement: S4 Table — (PDF) [file pgen.1008494.s005.pdf]

S4 Table. Primers used in this work.

Primer name	Sequence 5’→3’	
**Primers used for gene disruption**		
REV3_UPTEF	CAATACAAAACTACAAGTTGTGGCGAAATAAAATGTTTGGAAATGAGATCTGTTTAGCTTGCC	
REV3_DNTEF	ATAACTACTCATCATTTTGCGAGACATATCTGTGTCTAGATTATTCGAGCTCGTTTTCGACAC	
RAD52_UPTEF	ACGAAAAATATAGCGGCGGGCGGGTTACGCGACCGGTATCGAATGGAGATCTGTTTAGCTTGCC	
RAD52_DNTEF	ATAATGATGCAAATTTTTTATTTGTTTCGGCCAGGAAGCGTTTCAATTCGAGCTCGTTTTCGACAC	
MSH2_UPTEF	CTTTATCTGCTGACCTAACATCAAAATCCTCAGATTAAAAGTATGAGATCTGTTTAGCTTGCC	
MSH2_DNTEF	ATTATCTATCGATTCTCACTTAAGATGTCGTTGTAATATTAATTATTCGAGCTCGTTTTCGACAC	
RAD51_UPTEF	ACGTAGTTATTTGTTAAAGGCCTACTAATTTGTTATCGTCATATGGAGATCTGTTTAGCTTGCC	
RAD51_DNTEF	AAGTAAACCTGTGTAAATAAATAGAGACAAGAGACCAAATACCTAATTCGAGCTCGTTTTCGACAC	
MMS2_UPTEF	ATTCTGTATATGCAACGTAGAAGAAAGCAGCGTTTACACAAAAATGAGATCTGTTTAGCTTGCC	
MMS2_DNTEF	TGGCTTGGAATGCTGCAAATACTGTTTAGGAAAAAGTAGATAACTATTCGAGCTCGTTTTCGACAC	
PIF1_UPTEF	TTATCCATTGAGCGATTAGCTTACTTGTATCAATCAATTTTACATGAGATCTGTTTAGCTTGCC	
PIF1_DNTEF	ATAGCAGTTTGTATTCTATATAACTATGTGTATTAATATGTACTTATTCGAGCTCGTTTTCGACAC	
POL32_UPTEF	ATAATATTTCACATTAACTAACAACCAGAAATAGGCTTTAGTTAACTCAATCGGTAATTA	
POL32_DNTEF	CATTTGTATTATACATTACATCACAATTAGTAATGGAAAGTGTTTGGAAAAAAAAGAAG	
**Primers used for deletion confirmation**		
HPH UO	ACAGACGTCGCGGTGAGTTCAG	
HPH DO	TCGCCGATAGTGGAAACCGACG	
NAT1 UO	ACCGGTAAGCCGTGTCGTCAAG	
NAT1 DO	GCTTCGTGGTCGTCTCGTACTC	
REV3 A	AATTCTGCCAATCTATTTGATCTTG	
REV3 B	TCTGATTTAGAGGATGATCTAACCG	
REV3 C	TAAATGAAGACCATAGAGCAGAACC	
REV3 D	CACCAGATAGAGTTTTGAACGAAAT	
RAD52 A	GATTCAACAACTCCCTTGGCGTC	
RAD52 B	CAACCTTCGATGTATGCAATCCTG	
RAD52 C	CGCGTGAAACCACACCAA	
RAD52 D	TACGACACATGGAGGAAAGAAAAAC	
RAD51 A	CCAATCTAGTTTAGCTATCCTGCAA	
RAD51 B	AAAGTGTGACATAGCTGGGACTTAC	
RAD51 C	GTAAGTCCCAGCTATGTCACACTTT	
RAD51 D	AATTTTTCTCTTCACTCCCCTAAAA	*Saccharomyces cerevisiae* Genome Deletion Project
MSH2 A	CGTATAAACAAAGCCAAAGACAAGT	
MSH2 B	CCCAATTGAATCAAGAAACTCTCTA	
MSH2 C	TGAATTGACAGAATTGTCTGAAAAA	
MSH2 D	ACATCTCTTGTTTATCCCATCCATA	
PIF1 A	AAAGGCGCGTCTTAATTTTCTTCACT	
PIF1 B	GTGCGATACGTTTTTGAGTAAAAGAAA	
PIF1 C	ATCAAGTTCATTCATTGGTTTCCGAC	
PIF1 D	CTTTTTTCTATCGAAGGAGGTTCACC	
MMS2 A	CACCACTATTGCTCATTTTGTACTG	
MMS2 B	TAATATCGTCGCTATCAGCTAAACC	
MMS2 C	AAGATAAATCTACCATGCGTCAATC	
MMS2 D	TATTTATTATTGGCTTGGACTGGAG	
POL32 A	AATTCTCGATCAGTATGCCTCAATA	
POL32 B	TTTGTCTAGAGGTTTCCTTGTCATC	
**Primers used for confirmation of *PSF1* or *psf1-1* presence**		
InProm	AGCTAGGTTCCAAGAAGGCT	
dwPSF1	CCAGCTTGAAAGCATCGATA	
**Primers used for verification of integration plasmids carrying trinucleotide tracts into the *Lys2* locus**		
Lys2A	TGTGCCTTTGTTACGTCTATATTCA	
Lys2D	GAAGCTTCGCAAGTATTCATTTTAG	
TRP1A	AGAGACCAATCAGTAAAAATC	
TRP1D	GCGAAAAGACGATAAATACAA	
URA3UP	GGAGCACAGACTTAGATTGG	
URA3LW	CGAGATTCCCGGGTAATAAC	

Primers used for measurement of the stability of the trinucleotide tract	
OBL157	AGCAACAGGACTAGGATGAGTAGC
Tri1S	ACTTGGGGAGAGGTGCG
Tri1S2	TGCTTTGCCGATGTTACTTGGG
TNRUP1	CGGCTTTCAAGACACCCATC
TNRUP2	TGTAACGTTCACCCTCTACC
TNRUP3	GCCCTTGCATGACAATTCTG
TNRUP4	GGGACCTAATGCTTCAAC
**Primers used for measurement of the stability of repetitive sequences located on plasmids**	
GT_FOR	GTGGTTAGCAATCGCCTTAC
GT_REV	GTTTTCCCAGTCACGAC
REP1_FAM	6-FAM-CACGACGTTGTAAAACGAC
REP2_ROX	ROX-CACGACGTTGTAAAACGAC
REP3_HEX	HEX-CACGACGTTGTAAAACGAC
REP4_TAMRA	TAMRA-CACGACGTTGTAAAACGAC
**Primers used for construction of the *RFA1-YFP* fusion cassette**	
RFA7317F	CAATCGGCTGCTAGCTTAAC
RFA6231R	ACGGTTCACAATCCCTACAG
LEU2_F_H	ACCAAGCTTCGACCCTCGAGGAGAACTTCTAG
LEU2_R_H	GCAAAGCTTCGACTACGTCGTAAGGCCGTTTC
