# Supplementary material for: Defects in the GINS complex increase the instability of repetitive sequences via a recombination-dependent mechanism
Source: PLoS Genet. 2019 Dec 9;15(12):e1008494. doi: 10.1371/journal.pgen.1008494 (PMC6922473; doi:10.1371/journal.pgen.1008494)
Supplement: S5 Table — (PDF) [file pgen.1008494.s006.pdf]

S5 Table. Random sequences used in control experiments.

**Chromosomal TNR expansion assay**
CCCAGGTCGCCGTCGTCCCCGTACGCGACGAACGTCCGGGAGTCCGGGTCGCCGTCCTCCCCGTCGTCCGATTCG
**Plasmid-based frame shift assay**
GTCGACATGCGCTGGCCGCTTGCGTTGCGTCGTTGCTCTTTCTCGAG
